# A single intra‐articular stromal vascular fraction with platelet‐rich plasma injection yields superior clinical outcomes than a hyaluronic acid injection in patients with knee osteoarthritis: A prospective comparative study

**DOI:** 10.1002/ksa.70068

**Published:** 2025-09-12

**Authors:** Trifon Totlis, Vlasios Achlatis, Panagiotis‐Konstantinos Emfietzis, Theodorakys Marín Fermín, Theodoros Pettas, Aristotelis Sideridis, Ioannis Terzidis

**Affiliations:** ^1^ School of Medicine, Faculty of Health Sciences Aristotle University of Thessaloniki Thessaloniki Greece; ^2^ Thessaloniki Minimally Invasive Surgery (The‐MIS) Orthopaedic Centre St. Luke's Hospital Thessaloniki Greece; ^3^ Clínica Santa Sofía, Av. Principal de Santa Sofía, El Cafetal Caracas Venezuela

**Keywords:** adipose‐derived stem cells, hyaluronic acid, knee osteoarthritis, platelet rich plasma, stromal vascular fraction

## Abstract

**Purpose:**

The present study aimed to compare the efficacy and safety of a combined injection of stromal vascular fraction (SVF) and platelet rich plasma (PRP) versus a high molecular weight (HMW) hyaluronic acid (HA) injection in patients with knee osteoarthritis (KOA).

**Methods:**

A prospective comparative analysis was conducted for patients with KOA who underwent either a single intra‐articular mechanical SVF combined with PRP injection (Group A) or a single intra‐articular injection of HMW HA (Group B). The Knee Injury and Osteoarthritis Outcome Score (KOOS), the visual analogue scale (VAS) and the EuroQol EQ‐5D (EQ‐5D‐5L) were assessed at baseline, 3, 6 and 12 months postinjection. PRP was coded according to the DEPA classification.

**Results:**

The study included 108 knees from 67 patients (Group A: 31; Group B: 36). The VAS, KOOS total and EQ‐5D‐5L scores significantly improved within each group in every timepoint compared to baseline. The SVF‐PRP group significantly outperformed the HMW‐HA group in VAS, KOOS total and EQ‐5D‐5L scores at 6 months and 12 months follow‐up. The proportion of patients who achieved Minimal Clinically Important Difference (MCID) at 12 months was 87.0% versus 57.4% (*p* = 0.0003) in KOOS, 74.1% versus 61.1% in VAS (*p* = 0.152) and 64.8% versus 40.7% (*p* = 0.005) in EQ‐5D‐5L, for the SVF‐PRP vs HA group, respectively. No serious adverse events were reported in either group. Minor local adverse events were more common in the SVF‐PRP group and spontaneously resolved within days. The implemented PRP was coded as CCA.

**Conclusion:**

Both SVF‐PRP and HA injections are safe treatments, with no serious adverse events, and significantly improve pain, function and quality of life in patients with KOA. The SVF‐PRP outperformed the HA group in all three PROMs at 6 months and 12 months follow‐up, and in the proportion of patients who achieved MCID at 12 months postinjection.

**Level of Evidence:**

Level II.

**Abbreviations**
0 m: baseline
12 m: 12 months
3 m: 3 months
6 m: 6 months
ADSCs: adipose‐derived stem cells
CBC: complete blood count
DEPA: Dose of injected Platelets, Efficiency of production, Purity of the PRP, Activation of the PRP
EQ‐5D‐5L: EuroQuality of Life – 5 Dimensions – 5 Levels
HA: hyaluronic Acid
Hb: haemoglobin
HMW: high molecular weight
Ht: haematocrit
IQR: interquartile range
IRB: Institutional Review Board
KOA: knee osteoarthritis
KOOS: Knee injury and Osteoarthritis Outcome Score
K‐L: Kellgren–Lawrence
MCID: minimum clinically important difference
MIBO: Minimum Information for studies evaluating Biologics in Orthopaedics
MOCART: Magnetic resonance Observation of Cartilage Repair Tissue
MRI: magnetic resonance imaging
MSCs: mesenchymal stem cells
NSAIDs: non‐steroidal anti‐inflammatory drugs
OA: osteoarthritis
PASS: Patient Acceptable Symptom State
PROMs: Patient Reported Outcome Measures
PRP: platelet‐rich‐plasma
RBC: red blood cells
RCT: randomised controlled trial
rt‐PCR: real time polymerase chain reaction
SD: standard deviation
SVF: stromal vascular fraction
tSVF: tissue SVF
VAS: Visual Analogue Scale
WBC: white blood cells
WOMAC: Western Ontario and McMaster Universities Osteoarthritis Index
WORMS: Whole‐Organ Magnetic Resonance Imaging Score

## INTRODUCTION

Orthobiologic injections for knee osteoarthritis (KOA), including platelet‐rich plasma (PRP), stromal vascular fraction (SVF), and bone marrow aspirate concentrate, have been incorporated into clinical practice aiming to reduce pain, improve function and modify the natural history of the disease [11, 14, 28, 39]. SVF is a heterogeneous product that contains not only adipose‐derived stem cells (ADSCs), but also macrophages, fibroblasts, pericytes, endothelial cells and other cell populations. This cellular heterogeneity could create high therapeutic potential due to its complementary mechanisms of action including immunomodulatory, anti‐inflammatory, proangiogenic, antiapoptotic and antifibrotic properties [9, 13, 20].

Several clinical studies have evaluated the outcomes of SVF injections in patients with KOA, demonstrating no or minor adverse events, and showed significantly improved patient reported outcome measures (PROMs) and encouraging signs of cartilage regeneration or no deterioration in posttreatment magnetic resonance imaging (MRI) [13, 19, 22, 26, 45]. A recent systematic review summarised the relevant literature and confirmed the safety and efficacy of SVF interventions, either alone or in combination with other orthobiologics, while also highlighting the low quality of existing evidence and the lack of prospective comparative studies [9]. This prospective study aimed to compare the clinical efficacy of a single intra‐articular injection of SVF combined with PRP versus a single intra‐articular injection of high molecular weight (HMW) hyaluronic acid (HA) in patients with KOA. The hypothesis was that the clinical outcome of SVF‐PRP treatment is significantly better than that of HMW HA.

## MATERIALS AND METHODS

### Study design

A prospective comparative analysis was conducted in St. Luke's Hospital in Thessaloniki, Greece, for KOA patients who underwent intra‐articular injections from 20 September 2021 to 15 May 2023, with previous approval by the Institutional Review Board (IRB) of the Scientific Committee at St. Luke's Hospital in Thessaloniki (6 August 2021). Two methods were compared: single intra‐articular SVF‐PRP injection and single intra‐articular injection of HMW HA. After being thoroughly informed about both treatment options, patients were prospectively enroled and allocated to either the SVF‐PRP group or the HA group based on shared decision‐making between the patient and the senior author. This study complied with the principles of the Helsinki Declaration, ensuring that patients' rights, safety, and well‐being were consistently prioritised, and was complied with the DEPA (Dose of injected platelets, Efficiency of production, Purity of the PRP, Activation of the PRP) and short‐MIBO (Minimum Information for studies evaluating Biologics in Orthopaedics) guidelines [30, 31].

### Study participants and eligibility criteria

Patients were eligible for SVF‐PRP or HMW‐HA if they fulfilled the following criteria: (1) were men or women between 18 and 80 years; (2) had unilateral or bilateral Kellgren–Lawrence (K‐L) IΙ–IV KOA confirmed by an X‐ray; (3) presented no axial deviation of the affected knee(s) >15° (varus or valgus); (4) had an initial pain level ≥4 on a 10‐point visual analogue scale (VAS); (5) demonstrated the ability to comprehend instructions provided by the medical staff; and (6) possessed sufficient body fat to undergo liposuction if assigned to the SVF‐PRP group.

Patients were excluded if: (1) had suffered a recent severe trauma to the knee; (2) had previous knee intra‐articular injectable therapy within the last three months; (3) underwent surgery on the affected knee <1 year ago; (4) had Grade IV osteoarthritis (OA) in joints other than the knee; (5) had signs of local or systemic infection; (6) had any malignancy, autoimmune disorder or immunosuppression; (7) were on anticoagulants, antiplatelet, antithrombotic, or non‐steroidal anti‐inflammatory drugs (NSAIDs) <7 days before the injection; (8) had systemic corticosteroids <14 days before; (9) were diagnosed with abdominal hernia; (10) had history of cancer affecting the musculoskeletal system; (11) reported a history of allergy to any substances used within the treatments; and/or (12) were pregnant or breastfeeding.

### Outcome measures

Patients' demographic data, medical history, radiographic KOA status, and VAS were documented before the intervention. Primary outcomes included the VAS, the Knee Injury and Osteoarthritis Outcome Score (KOOS) and subscores, and the EuroQol‐5D‐5L (EQ‐5D‐5L) questionnaires. Patient outcomes were assessed at baseline (0 m), 3 months (3 m), 6 months (6 m) and 12 months postinjection (12 m) by an independent investigator. These three PROMs were collected through in‐person assessments before the intervention (0 m) and subsequently via telephone. Based on previous publications, a minimum clinically important difference (MCID) of 10 points for the total KOOS score (0–100), 1.4 points for the VAS score (0–10) and 0.2 points for the EQ‐5D‐5L (0–1) were set as a reference value for clinical improvement. Additionally, a patient acceptable symptom state (PASS) of 64 points for the total KOOS score and 3.0 for VAS were set [10, 27, 48].

### Intervention

#### SVF‐PRP preparation technique

The current mechanical SVF (tSVF) preparation is a modified version of the technique reported by the manufacturers [5, 43]. The detailed protocol has been previously published [44]. The patient was positioned in a 30° beach chair position, and 5 mL local anaesthetic (lidocaine 2% with adrenaline 0.5%) was subcutaneously administered. A stab incision was made superomedial to the anterior superior iliac spine, and approximately 180 mL of a modified Klein solution was infiltrated in the ipsilateral iliac and hypogastric regions using the ACA‐Kit ABS‐10056 infiltration cannula (Arthrex GmbH, Munich, Germany). After 12 min, 60–70 mL of lipoaspirate was collected and processed through centrifugation and mechanical dissociation to obtain the tSVF.

Meanwhile, PRP was obtained using the manufacturer's standard protocol method which involves collecting 30 mL of venous blood from the patient's antecubital fossa with two ACP® Double‐Syringes (Arthrex GmbH, Munich, Germany). The venous blood, without any anticoagulants, was centrifuged at 1500 rpm (340*g*) for 5 min. The resulting 8–12 mL of PRP was then aspirated into the inner syringe of the double‐syringe system. The prepared PRP (8–12 mL) was transferred into a 20 mL luer‐lock syringe in liquid form without any activator agent and then emulsified with the tSVF by repeatedly passing the solution between two connected luer‐lock syringes via a 1.2 mm fractionator. This process reduced the viscosity of the solution to prevent needle obstruction. The final mixture was injected intra‐articularly into each knee via a lateral parapatellar approach, with 5–8 mL administered per knee, consisting of 1–2 mL of tSVF and 4–6 mL of PRP.

#### Laboratory analysis

From 21 of the patients assigned to the SVF‐PRP group, an additional 5 mL of venous blood was collected during the preparation of PRP. These samples were promptly sent to the laboratory for a complete blood count (CBC) analysis. The CBC included measurements of red blood cells (RBC), white blood cells (WBC) (subcategorised into neutrophils, lymphocytes, and monocytes), and platelets. Haemoglobin (Hb) and haematocrit (Ht) levels were also assessed. An additional laboratory analysis was performed to evaluate the quality of the PRP. From the PRP obtained from each patient, 1–2 mL was immediately sent for analysis. This analysis measured the concentration of platelets, RBCs, and WBCs, including neutrophils, lymphocytes, and monocytes. The efficiency and purity of the PRP preparation were assessed, and the platelet recovery rate was calculated and classified based on established literature [4, 30].

Furthermore, a portion of the tSVF solution from 23 patients of Group A was utilised to analyse its cellular composition through flow cytometry, while the gene expression profile from 16 of them was examined using real‐time polymerase chain reaction (rt‐PCR). The results of these analyses have been previously published [44].

#### HMW‐HA product

The OPTIVISC Single® (Biotech, Roscommon, Ireland) HA viscosupplementation product was used. The product contains HMW HA 3% (90 mg, 2.8–3.2 MDa) in a 3 mL pre‐filled sterile syringe. These pre‐filled syringes were individually sealed, placed in sterile packets, and autoclaved. Thus, both the contents and surfaces of the syringes were sterile.

#### Intraarticular injection technique and postprocedure care

All intraarticular injections were performed by the senior author, with the patient in a supine position using a lateral parapatellar approach under sterile conditions. Patients were advised to restrict their lower limb activity for at least 3–5 days after the intervention and apply cold therapy to the affected area. During the study, oral acetaminophen/paracetamol, was allowed with a maximum dosage of 3 g daily. Gradual resumption of daily activities was allowed 3–5 days postinjection. Both groups adhered to an identical 6‐week rehabilitation protocol, initiated one week following the intervention. The programme included progressive range‐of‐motion exercises, therapist‐assisted stretching, quadriceps activation and isometric strengthening. This was followed by a progressive introduction of closed kinetic chain exercises, resistance strengthening for the pelvic and lower limb muscles, as well as balance, proprioception, and gait training. Exercise progression was guided by pain tolerance, and regular sports or recreational activities were encouraged after a minimum of three weeks postinjection, as tolerated.

### Statistical analysis

The normality of distributions was explored with the Shapiro–Wilk test and skewness tests. Groups were compared to verify homogeneity using the independent samples *t*‐test, Mann–Whitney *U* test, or chi‐square test. Robustness checks and supplemental tests were performed when possible. Descriptive statistics were calculated for PROMs at each group's time point and compared using the parametric repeated measures analysis of variance test. If one or more parameters were abnormally distributed at one or more points, then the non‐parametric Mann–Whitney *U* test was implemented. An additional Friedman test was conducted to assess within‐group differences over time. If a statistically significant difference in a parameter over time was observed in one group, then Wilcoxon Signed Ranks tests were performed.

### Sample size calculation

To estimate an acceptable sample size, we used the following parameters: a 0.05 level of statistical significance (*α* = 0.05), a β‐error of 20% (*β* = 0.2) resulting in a statistical power of 80%, and indicative values of the main outcome measure (KOOS total) in patients with KOA before intervention (indicative mean value = 50.0), after intervention with HMW HA (indicative mean value = 60.0), and after intervention with SVF‐PRP (indicative mean value = 75.0) based on the literature [2, 6, 7, 16, 18]. Lastly, an indicative value of 17.0 points was considered for the standard deviation of KOOS total in patients with KOA before intervention [2, 6, 7]. The minimum proposed sample size calculated was 23 patients for each group, assuming a 1:1 ratio, totalling 46 patients.

## RESULTS

The study included 108 knees from 67 patients (Group A: 31 and Group B: 36), which exceeded the sufficient sample size based on our calculations. No statistically significant difference was found between groups at baseline (Table 1).

**Table 1:** Comparisons of the sample characteristics between the two groups.

	SVF‐PRP (Group A)	HMW HA (Group B)		
Sample	Group A	Group B		
Knees	*n* = 54	*n* = 54		
Patients	*n* = 31	*n* = 36		
Age	Group A mean (SD)	Group B mean (SD)	Primary comparison	Robustness check
	61.0 (7.9)	65.1 (9.0)	*p* = 0.055	*p* = 0.080
BMI	Group A mean (SD)	Group B mean (SD)	Primary comparison	Robustness check
	30.6 (3.8)	30.6 (3.2)	*p* = 0.958	*p* = 0.739
OA grade	Group A	Group B	Primary comparison	Robustness check
Grade II	*n* = 16	*n* = 18	*p* = 0.889	*Not needed*
Grade III	*n* = 20	*n* = 20		
Grade IV	*n* = 18	*n* = 16		
Parameter	Group A mean (SD) or median (IQR)	Group B mean (SD) or median (IQR)	Primary comparison	Robustness check
KOOS 0 m	52.8 (14.2)	50.5 (14.0)	*p* = 0.387	*p* = 0.398
VAS 0 m	6.1 (1.7)	6.4 (1.7)	*p* = 0.360	*p* = 0.460
EQ‐5D‐5L 0 m	0.539 (0.361)	0.539 (0.312)	*p* = 0.796	*Not needed*

Abbreviations: 0 m, baseline; BMI, body mass index; EQ‐5D‐5L, EuroQuality of Life–5 Dimensions–5 Levels; HA, hyaluronic acid; HMW, high molecular weight; KOOS, Knee injury and Osteoarthritis Outcome; PRP, platelet rich plasma; SVF, stromal vascular fraction; VAS, Visual Analogue Scale.

### PRP classification

Whole blood analysis revealed a mean platelet count of 241.3 ± 64.5 × 10^9^/L (95% confidence interval [CI] [213.41–269.19 × 10^9^/L]). The corresponding PRP samples demonstrated a mean platelet concentration of 477.4 ± 155.0 × 109/L (95% CI [410.37–544.43 × 10^9^/L]). The distribution of other blood cell populations is presented in Table 2a. According to the DEPA classification [30], the implemented PRP is a CCA.

**Table 2:** a. Results of the whole blood and supplementary PRP analyses performed for a subset of Group A patients. b. Results of the SVF flow cytometry analysis performed for a subset of Group A patients.

a.		
	Whole blood (*10^9^/L)	Platelet‐rich‐plasma (*10^9^/L)
WBC mean ± SD	6.9 ± 1.84	0.3 ± 0.2
RBC mean ± SD	4529 ± 396	17.6 ± 14.1
PLT mean ± SD	241.3 ± 64.5	477.4 ± 155.0

b.	
SVF flow cytometry analysis	Cells count (*10^6^/mL)
CD90^+^/CD105^+^ ADSC mean ± SD	44.5 ± 23.2
HSC mean ± SD	0.5 ± 0.2
Pericytes mean ± SD	2.7 ± 1.2
Endothelial cells mean ± SD	1.9 ± 0.6
Total viable nucleated cells mean ± SD	49.7 ± 22.9

Abbreviations: ADSC, adipose‐derived stem cells; HSC, haemopoietic stem cells; PLT, platelets; PRP, platelet rich plasma; RBC, red blood cells; SD, standard deviation; SVF, stromal vascular fraction; WBC, white blood cells.

### SVF composition

Flow cytometry analysis of the SVF product revealed a mean concentration of 49.7 × 10⁶ (95% CI [39.8–59.6 × 10⁶/mL]) viable nucleated cells per mL. Among these, an average of 44.5 × 10⁶ (95% CI [34.5–54.5 × 10⁶/mL]) were identified as CD90⁺/CD105⁺ ADSCs. The distribution of additional cell populations is detailed in Table 2b.

### Postinjection scores

**Figure 1:**
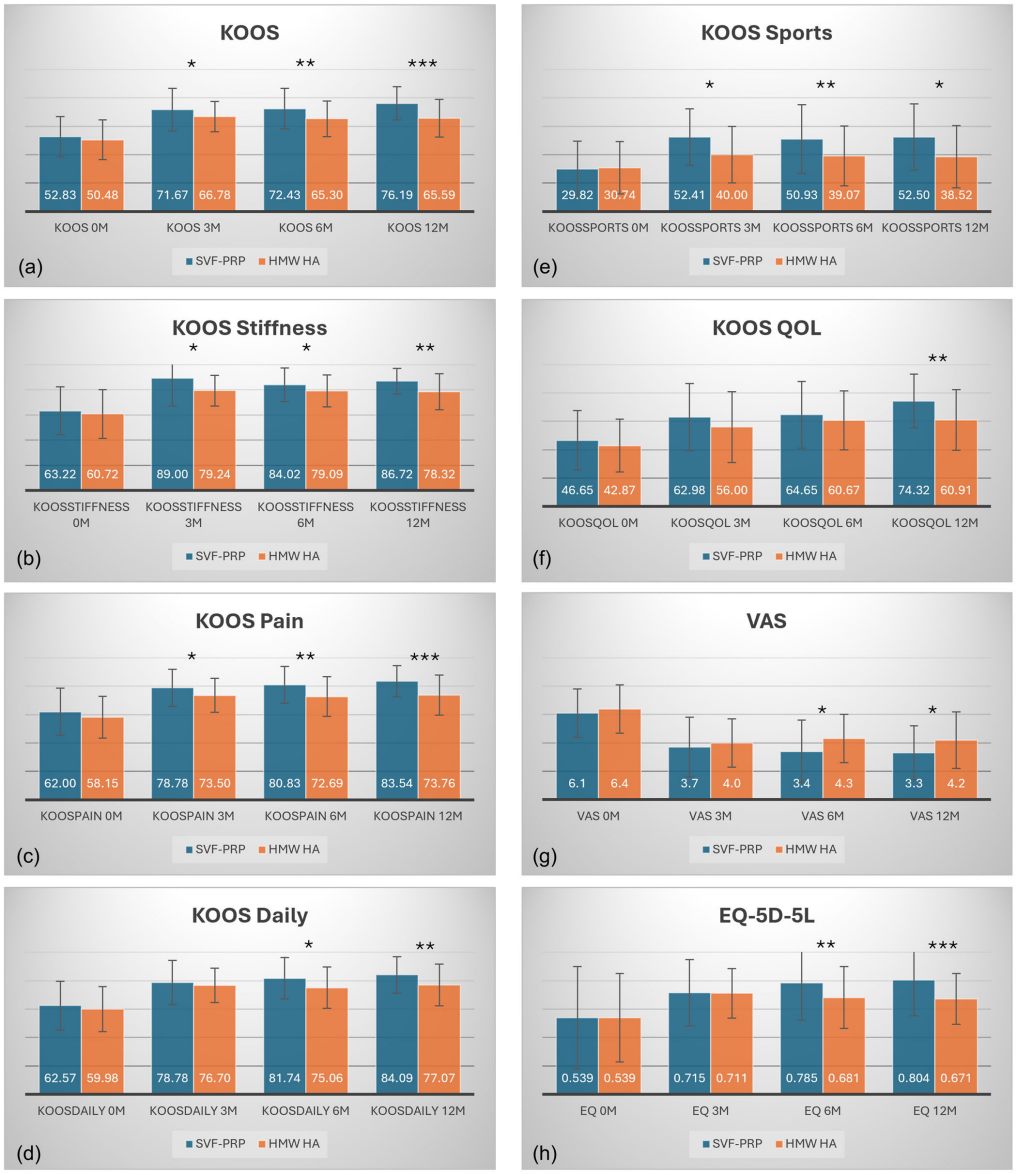
Graphical depiction of the comparisons of Patient Reported Outcome Measures’ means (or medians) between the two groups in all available timepoints (*p < 0.05; **p < 0.01; ***p < 0.001). (a) KOOS total. (b) KOOS stiffness. (c) KOOS pain. (d) KOOS activities of daily living. (e) KOOS sports. (f) KOOS quality of life. (g) VAS. (h) EQ‐5D‐5L. EQ‐5D‐5L, EuroQuality of Life–5 Dimensions–5 Levels; KOOS, Knee injury and Osteoarthritis Outcome Score; VAS, Visual Analogue Scale.

The VAS, KOOS total and EQ‐5D‐5L scores significantly improved within each group at every time point compared to baseline (*p* < 0.05). The SVF‐PRP group significantly outperformed the HMW‐HA group in VAS, KOOS total, and EQ‐5D‐5L scores at 6 m and 12 m follow‐up postinjection (*p* < 0.05). The VAS and EQ‐5D‐5L differences between the groups were non‐significant at 3 m, but were significant in KOOS total (*p* < 0.05) favouring the SVF‐PRP group (Figure 1 and Table 3).

**Table 3:** Comparisons of Patient Reported Outcome Measures' means (or medians) between the two groups in all available timepoints.

	SVF‐PRP (Group A)	HMW HA (Group B)		
Parameters	Mean (SD) or median (IQR)	Mean (SD) or median (IQR)	Primary comparison	Robustness check
KOOS				
KOOS 0 m	52.8 (14.2)	50.5 (14.0)	*p* = 0.387	*p* = 0.398
KOOS 3 m	71.7 (15.0)	66.8 (10.7)	*p* = 0.054	*p* = 0.039*
KOOS 6 m	72.4 (14.4)	65.3 (12.6)	*p* = 0.007**	*p* = 0.007**
KOOS 12 m	76.2 (11.7)	65.6 (13.3)	*p* < 0.001***	*p* < 0.001***
KOOS stiffness				
KOOS stiffness 0 m	63.2 (19.1)	60.7 (19.2)	*p* = 0.473	Not needed
KOOS stiffness 3 m	89.0 (22.0)	79.2 (12.1)	*p* = 0.029*	Not needed
KOOS stiffness 6 m	84.0 (13.3)	79.1 (12.8)	*p* = 0.034*	Not needed
KOOS stiffness 12 m	86.7 (10.4)	78.3 (14.3)	*p* = 0.001**	Not needed
KOOS pain				
KOOS pain 0 m	62.0 (16.7)	58.2 (14.8)	*p* = 0.206	*p* = 0.136
KOOS pain 3 m	78.8 (13.1)	73.5 (12.0)	*p* = 0.031*	*p* = 0.022*
KOOS pain 6 m	80.8 (13.0)	72.7 (13.9)	*p* = 0.002**	*p* = 0.002**
KOOS pain 12 m	83.5 (11.0)	73.8 (14.1)	*p* < 0.001***	*p* < 0.001***
KOOS daily				
KOOS daily 0 m	62.6 (17.2)	60.0 (15.8)	*p* = 0.416	*p* = 0.322
KOOS daily 3 m	78.8 (15.5)	76.7 (12.2)	*p* = 0.442	*p* = 0.293
KOOS daily 6 m	81.7 (14.6)	75.1 (14.5)	*p* = 0.019*	*p* = 0.014*
KOOS daily 12 m	84.1 (12.8)	77.1 (14.6)	*p* = 0.009**	*p* = 0.009**
KOOS sports				
KOOS sports 0 m	29.8 (19.6)	30.7 (18.5)	*p* = 0.865	Not needed
KOOS sports 3 m	52.4 (20.0)	40.0 (20.0)	*p* = 0.043*	Not needed
KOOS sports 6 m	50.9 (24.2)	39.1 (21.1)	*p* = 0.009**	Not needed
KOOS sports 12 m	52.5 (23.4)	38.5 (21.8)	*p* = 0.010*	Not needed
KOOS QOL				
KOOS QOL 0 m	46.7 (20.9)	42.9 (18.6)	*p* = 0.387	Not needed
KOOS QOL 3 m	63.0 (23.7)	56.0 (25.0)	*p* = 0.586	Not needed
KOOS QOL 6 m	64.7 (23.5)	60.7 (20.7)	*p* = 0.176	Not needed
KOOS QOL 12 m	74.3 (18.9)	60.9 (21.4)	*p* = 0.001**	Not needed
VAS				
VAS 0 m	6.1 (1.7)	6.4 (1.7)	*p* = 0.360	*p* = 0.460
VAS 3 m	3.7 (2.1)	4.0 (1.7)	*p* = 0.445	*p* = 0.306
VAS 6 m	3.4 (2.2)	4.3 (1.7)	*p* = 0.031*	*p* = 0.021*
VAS 12 m	3.3 (1.9)	4.2 (2.0)	*p* = 0.017*	*p* = 0.028*
EQ‐5D‐5L				
EQ‐5D‐5L 0 m	0.539 (0.361)	0.539 (0.312)	*p* = 0.796	Not needed
EQ‐5D‐5L 3 m	0.715 (0.233)	0.711 (0.175)	*p* = 0.248	Not needed
EQ‐5D‐5L 6 m	0.785 (0.264)	0.681 (0.218)	*p* = 0.004**	Not needed
EQ‐5D‐5L 12 m	0.804 (0.249)	0.671 (0.179)	*p* < 0.001***	Not needed

Abbreviations: 0 m, baseline; 3 m, 3 months; 6 m, 6 months; 12 m, 12 months; EQ‐5D‐5L, EuroQuality of Life – 5 Dimensions – 5 Levels; HA, hyaluronic acid; HMW, high molecular weight; IQR, interquartile range; KOOS, Knee injury and Osteoarthritis Outcome Score; PRP, platelet rich plasma; QOL, quality of life; SD, standard deviation; SVF, stromal vascular fraction; VAS, visual analogue scale.

* *p* < 0.05;

** *p* < 0.01;

*** *p* < 0.001.

### MCID and PASS

A significantly higher proportion of patients in the SVF‐PRP group compared with the HA group achieved PASS in KOOS (74.1% vs. 55.6%, *p* = 0.031) and VAS (57.4% vs. 37.0%, *p* = 0.017) at 6 m. Similarly, a statistically significantly higher percentage of patients undergoing SVF‐PRP had MCID improvement in KOOS (87.0% vs. 57.4%, *p* = 0.0003) and EQ‐5D‐5L (64.8% vs. 40.7%, *p* = 0.005), and achieved PASS in KOOS (81.5% vs. 55.6%, *p* = 0.0022) at 12 m. A higher proportion of patients in the SVF‐PRP group achieved both MCID improvement (74.1% vs. 61.1%, *p* = 0.152) and PASS (51.9% vs. 44.4%, *p* = 0.443) in VAS score compared to the HA group at 12 m, but these differences did not reach statistical significance (Table 4).

**Table 4:** Percentage of cases achieving MCID and PASS from both groups at 6 and 12 months after treatment.

		MCID		PASS	
		SVF‐PRP	HA	SVF‐PRP	HA
6 months after treatment	KOOS	75.9%	68.5%	74.1%	55.6%
	VAS	75.9%	74.1%	57.4%	37.0%
	EQ‐5D‐5L	64.8%	48.2%	‐	‐
12 months after treatment	KOOS	87.0%	57.4%	81.5%	55.6%
	VAS	74.1%	61.1%	51.9%	44.4%
	EQ‐5D‐5L	64.8%	40.7%	‐	‐

Abbreviations: EQ‐5D‐5L, EuroQuality of Life – 5 Dimensions – 5 Levels; HA, hyaluronic acid; HMW, high molecular weight; KOOS, Knee injury and Osteoarthritis Outcome Score; MCID, minimum clinically important difference; PASS, Patient Acceptable Symptom State; PRP, platelet rich plasma; SVF, stromal vascular fraction; VAS, Visual Analogue Scale.

### Adverse events

No serious adverse events were reported in either group. Three patients in the SVF‐PRP and two in the HA group reported a minor headache that was treated with paracetamol. This was not considered related to the intervention. In the SVF‐PRP group, fifteen patients reported redness (haematoma) and sensitivity, while seven patients only experienced sensitivity in the harvesting areas (iliac and hypogastric region). All of them were spontaneously resolved within 10 days after the intervention. Sixteen patients undergoing HA injection complained about discomfort or pain in the knee, which was spontaneously resolved within 1–3 days. All 31 patients in the SVF‐PRP group reported discomfort or pain in the knee, which typically resolved within 1–6 days, allowing them to feel the same as before the injection. All adverse events were considered mild and required no additional intervention beyond the standard postinjection cold therapy prescribed to all patients.

## DISCUSSION

The most important finding of the current study is that intra‐articular injection of SVF‐PRP, compared to HA, showed better KOOS total scores and their subsections (except for the KOOS quality of life subscore), as well as VAS and EQ‐5D‐5L scores at 6 months and 12 months, in patients with KOA. Furthermore, the proportion of patients who achieved PASS and MCID in the SVF‐PRP group was consistently higher than that of the HA group at all time points and PROMs, with the difference being more pronounced in the KOOS score and at the 12 months follow‐up (except for PASS in the VAS score).

Although the literature comparing SVF to HA is scarce, existing comparative studies demonstrate the superiority of the SVF, in line with the current cohort. Hong et al., in their randomised controlled trial (RCT), reported significant improvements in VAS, Western Ontario and McMaster Universities Osteoarthritis Index (WOMAC) scores, and range of motion (ROM) at 1‐year follow‐up compared to HA in 16 patients with bilateral KOA [22]. Moreover, whole‐organ magnetic resonance imaging score (WORMS) and magnetic resonance observation of cartilage repair tissue (MOCART) measurements revealed a significant articular cartilage improvement in the SVF group compared to HA. In another RCT including 126 KOA patients, the authors reported acceptable clinical outcomes in approximately 60% of patients up to 5 years after SVF treatment, with significantly better VAS and WOMAC scores, as well as total cartilage volume, in the SVF group compared to the HA group [50].

Orthobiologic injections combining two injectable agents are a common practice in KOA management, which has the potential to benefit from a synergistic effect [23, 24]. In the current study, SVF was combined with PRP. Thereby, this intervention transferred both autologous cells and autologous growth factors into the knee joint. Recently, Klingenberg et al. published a retrospective study on 213 patients, with moderate to severe KOA, who underwent one (41.8%) or two (50.2%) SVF‐PRP injections into the infra‐ or suprapatellar fat pad [26]. They revealed significant VAS and WOMAC pain reduction during a 2‐year follow‐up. Similarly, KOOS daily living activities and sports scores improved by 21% and 45%, respectively, during the same period and achieved MCID. A small comparative case series of 12 patients with K–L Grade III and IV KOA, treated with intra‐articular injections of SVF with and without PRP, reported 67% patient satisfaction at 1‐year follow‐up. Only patients with Grade IV KOA in the SVF without PRP treatment group reported 100% dissatisfaction [42]. Still, whether adding PRP to an SVF injection is beneficial for patients with KOA is unclear. In a systematic review, SVF was combined with PRP in eight studies and with fibrin in five studies. These studies demonstrated favourable outcomes when SVF and an orthobiologic were used [23]. The existing literature comparing SVF combined with PRP to SVF alone is very limited. Carvalho Schweich‐Adami et al. reported that cultivated ADSCs combined with PRP injection were superior to either cultivated ADSCs or PRP alone in patients with KOA [12]. Louis et al. found that microfat injections may provide significant clinical improvement in KOA patients, either with or without the use of PRP [29].

Basic science studies have demonstrated that PRP promotes MSCs proliferation and migration and delays the onset of the senescence phenotype [41]. Furthermore, adding PRP increases the volume of the SVF injection, facilitating its sufficient spread through all joint structures, especially in larger knees. Given that PRP has been shown to be superior to HA injections in patients with KOA [18, 36, 37], another question arises as to whether the clinical benefits observed with combined SVF‐PRP therapy can be attributed primarily to SVF or to PRP. This distinction remains unclear, as comparative studies directly evaluating SVF‐PRP versus PRP alone are limited in the current literature. The present study aimed to evaluate the clinical efficacy of a specific orthobiologic formulation combining SVF with PRP compared to HA injections.

In the present study, no significant difference was observed between the two treatment groups in any PROMs at 3 m postinjection. The SVF‐PRP significantly outperformed HA at 6 m and the difference became more prominent at 12 m, as shown in Table 3 and Figure 1. Only a few articles have addressed the long‐term outcomes of SVF in patients with KOA. At a 5‐year follow‐up, Zhang et al. reported sustained improvements in two‐thirds of the patients, in terms of pain, function, and quality of life compared to baseline measurements, highlighting SVF's long‐term therapeutic potential [50]. On the other hand, a recently published single‐arm study in 20 patients with K‐L Grade 2–3 KOA, revealed significant improvements in pain and function in the first 2 years following an SVF injection, which disappeared at 3 years postinjection [13].

Direct comparisons to other studies are challenging due to the heterogeneity in KOA classifications, implemented PROMs, follow‐up time points, and fat harvesting techniques [2, 20]. Furthermore, the inconsistent reporting of SVF and PRP preparation protocols, as well as the frequent lack of comprehensive characterisation of the final products, creates significant challenges for comparability across research studies [32, 40]. The composition of the SVF‐PRP used in the current cohort has been comprehensively characterised, with a mean concentration of CD90⁺/CD105⁺ ADSCs of 44.5 × 10⁶ cells/mL [44] and a platelet recovery rate of approximately twice the baseline peripheral blood platelet count. Therefore, an injection volume of 1–2 mL corresponds to the administration of 44.5–89.0 × 10⁶ ADSCs into the joint, a cell count that falls within the range commonly reported in the relevant literature [13, 34]. The orthopaedic scientific community is still investigating the ideal orthobiologics dose, as cumulative evidence shows a dose‐dependent effect [8, 15, 19, 33, 45, 46, 47]. Notably, studies using expanded ADSCs have indicated that patients receiving high concentrations of stem cells experienced significantly greater clinical improvements than those with low and medium concentrations [1, 25, 49]. Other studies have diverged from the previously mentioned findings, reporting that the group receiving the lowest concentration exhibited better clinical outcomes [21, 38].

A higher BMI has been associated with increased pro‐inflammatory cytokine expression in adipose tissue, which may potentially affect the biological quality and therapeutic efficacy of adipose‐derived cell products [35]. In the present study, the mean BMI of both treatment groups was similarly elevated, averaging approximately 30.6 kg/m². However, biochemical analysis of the SVF product used in this study revealed no significant correlation between BMI and the expression of the pro‐inflammatory cytokine interleukin‐1 (IL‐1) [44], suggesting that BMI may not have markedly impacted the inflammatory profile of the injected SVF in this cohort.

An encouraging observation across all relevant studies is the safety of this intervention [9, 20, 49], as further supported by the present findings. The most commonly reported adverse events are minor and transient, including mild abdominal pain, discomfort, ecchymosis following abdominal liposuction, and knee pain or swelling, which spontaneously resolve [9, 20]. The transient discomfort or knee pain following the SVF‐PRP injection was expected according to previous similar cases (before initiation of the study). Thereby, prior to intervention, all patients were informed about the potential adverse events and were advised to apply ice to the affected area postinjection.

The findings of the present study should be interpreted in light of the following limitations. The study's non‐randomised design may have led to selection bias, potentially influencing the allocation of groups. However, the sample size exceeded the number estimated during the a priori power analysis, and no significant differences were observed in baseline characteristics between the two groups. Additionally, results were not reported based on patients' K‐L grades, which hampers, as in other studies [3], the possibility of providing specific treatment recommendations. Furthermore, the methodology did not include any radiologic or MRI assessment following the intervention to assess potential disease‐modifying effects. Another limitation is the inherently higher invasiveness and cost associated with the SVF‐PRP treatment compared to HA injections. Although the clinical efficacy was objectively assessed in both groups, these factors may influence patient expectations, thereby introducing potential bias. Future efforts should aim for long‐term prospective RCTs comparing SVF‐PRP with multiple control groups, including SVF, PRP and HA. Such studies should incorporate MRI‐based assessments and ensure rigorous adherence to standardised reporting guidelines for orthobiologic procedures.

There is growing evidence about the positive effect of SVF on symptoms, function and cartilage degeneration in KOA patients. However, there is a paucity of comparative studies, which are crucial for evaluating the placebo effect and whether SVF is better than other traditional and less expensive injections. Two existing studies reported that SVF outperformed HA injections in VAS, WOMAC scores, and cartilage status, at 1‐year [22] and 5‐year follow‐up [50]. The findings of the present study strengthen these observations, adding to the superiority of SVF in the KOOS scale, quality of life improvement and the percentage of patients who achieved PASS and MCID. The recent ESSKA Consensus on cell‐based orthobiologics, does not recommend cell therapies as a first line injectable treatment for KOA. Cell treatments should be considered when other non‐operative and other injectable measures have failed and in circumstances where surgery is not yet mandated or not feasible [17]. Considering the aforementioned limitations, the current study contributes to the relevant literature by establishing SVF injections as a safe and reliable treatment option that may benefit patients who have not found relief after first‐line KOA treatment strategies, such as HA injections.

## CONCLUSION

Both SVF‐PRP and HA injections significantly improved pain, function and quality of life in patients with KOA. The SVF‐PRP outperformed the HA group in all three PROMs at 6 months and 12 months follow‐up, and in the proportion of patients who achieved PASS and MCID, especially at 12 months postinjection. No serious adverse events were reported in either group. Minor local adverse events were more common in the SVF‐PRP group, as expected due to the interventions, and spontaneously resolved within days.

## AUTHOR CONTRIBUTIONS

*Conceptualization*: Trifon Totlis, Aristotelis Sideridis and Ioannis Terzidis. *Methodology*: Trifon Totlis, Vlasios Achlatis, Panagiotis Emfietzis and Theodorakys Marín Fermín. *Formal analysis and investigation*: Trifon Totlis, Vlasios Achlatis, Panagiotis Emfietzis and Theodoros Pettas. *Writing–original draft preparation*: Vlasios Achlatis and Panagiotis Emfietzis. *Writing–review and editing*: Trifon Totlis and Theodorakys Marín Fermín. *Resources*: Trifon Totlis, Aristotelis Sideridis and Ioannis Terzidis. *Supervision*: Trifon Totlis and Ioannis Terzidis.

## CONFLICT OF INTEREST STATEMENT

The authors declare no conflicts of interest.

## ETHICS STATEMENT

Study received approval from the IRB of St. Luke's Clinic, Thessaloniki, Greece on 6 August 2021. This study complied with the principles of the Helsinki Declaration, ensuring that patients' rights, safety, and well‐being were consistently prioritised. Patients indicated their consent to participate in the study by signing an Informed Consent Form.

## Data Availability

Raw research data are available upon request.
